# Moving in a Moving World: A Review on Vestibular Motion Sickness

**DOI:** 10.3389/fneur.2016.00014

**Published:** 2016-02-15

**Authors:** Giovanni Bertolini, Dominik Straumann

**Affiliations:** ^1^Department of Neurology, University Hospital Zurich, Zurich, Switzerland

**Keywords:** motion sickness, vestibular, sensory conflict, cross-coupling stimulus, linear oscillations

## Abstract

Motion sickness is a common disturbance occurring in healthy people as a physiological response to exposure to motion stimuli that are unexpected on the basis of previous experience. The motion can be either real, and therefore perceived by the vestibular system, or illusory, as in the case of visual illusion. A multitude of studies has been performed in the last decades, substantiating different nauseogenic stimuli, studying their specific characteristics, proposing unifying theories, and testing possible countermeasures. Several reviews focused on one of these aspects; however, the link between specific nauseogenic stimuli and the unifying theories and models is often not clearly detailed. Readers unfamiliar with the topic, but studying a condition that may involve motion sickness, can therefore have difficulties to understand why a specific stimulus will induce motion sickness. So far, this general audience struggles to take advantage of the solid basis provided by existing theories and models. This review focuses on vestibular-only motion sickness, listing the relevant motion stimuli, clarifying the sensory signals involved, and framing them in the context of the current theories.

## Introduction

Motion sickness (1, 2) is a syndrome elicited in healthy subjects by ongoing passive self-motion that contains certain dynamic and kinematic properties (see Properties of Nauseogenic Stimuli). Illusions of such passive self-motion, as induced by moving visual surrounds, may also produce this condition (2). The first considerations on motion sickness known to us date back more than 2000 years, when the Greek physician Hippocrates observed that “sailing on the seas proves that motion disorders the body.” In those ancient times, boats provided one of the only few forms of passive motion to which individuals were exposed. Because passive motion (car, bus, train, and plane) and illusion of passive motion (video games on large screens, 3D movies, and virtual reality) are now abundant in modern life, motion sickness has become a frequent problem (3–8). As the time spent on transport systems occupies a considerable part of daily life, travelers normally perform a variety of activities while being transported, leading to various active head movements during passive motion. However, motion sickness can be provoked or aggravated by active head movements in the presence of passive motion (8–12), considerably hindering the quality of travel.

Depending on its severity, the syndrome of motion sickness consists of various combinations of the following signs and symptoms: drowsiness, dizziness, discomfort, restiveness, repetitive yawning, stomach awareness, nausea, pallor, sweating, headache, malaise, bradycardia, arterial hypotension vomiting, and apathy (2, 12, 13). Susceptibility to motion sickness varies considerably among subjects (14–19), whereby genetic factors and age play an important role (20, 21). Notably, there is a strong association between the susceptibility to motion sickness and migraine (22–29).

## Properties of Nauseogenic Stimuli

Sine qua non for developing motion sickness is exposure to a real or illusory motion stimulus (2). Subject without labyrinthine vestibular function do not become motion sick (30–32); thus, the vestibular system appears to always take part in a nauseogenic stimulus. One may argue, however, that motion sickness can also be caused by stimuli that do not activate the labyrinth such as visual illusion of motion (8, 33–35). To understand the link between these two apparently distinct provocative stimuli, it is important to consider that the vestibular system is constantly involved in the perception of self-motion (36), as the brain continuously takes the vestibular input into account. Overall, it is possible to assert that motion sickness is occurring whenever the subjects are exposed to stimuli causing conflicts between motion-sensitive input signals (2, 13).

**Figure 1:**
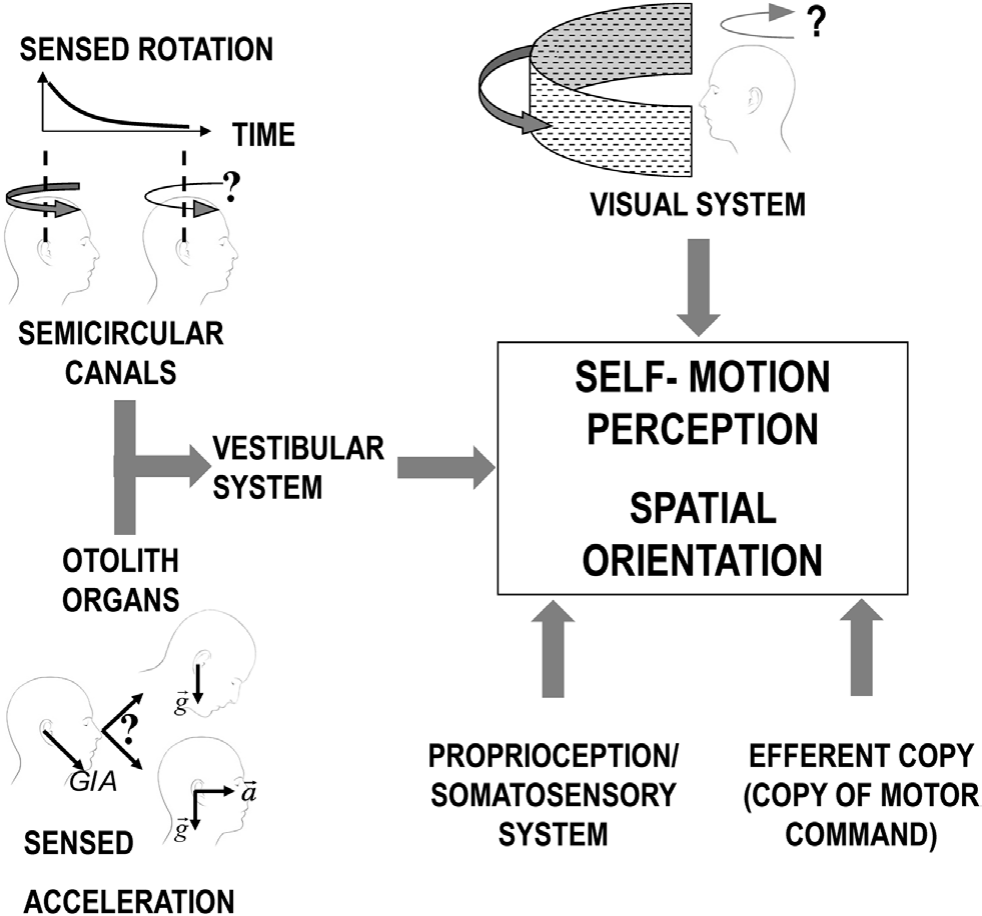
**Scheme of the different sensory systems contributing to the perception of self-motion**. For the vestibular system, the otolith organs and the semicircular canals are represented separated to allow evidencing the inherent limitations of the signal from each sensor before central processing. Specifically, the semicircular canals signal in response to continuous rotation decline over time (solid black line decaying in the plot in the top/left corner) leading to a similar decrease in the sensation of self-rotation (as depicted by the change in the curve arrow above the two heads). The otolith organs, instead, perceive only the overall GIA vector (sum of gravity and inertial acceleration) and cannot, therefore, distinguish between a head tilt and linear translation.

The motion-sensitive inputs to our nervous system originate from different sensory systems (mainly vestibular, but also visual and somatosensory). Each system has its sensory-specific sensitivity, optimized to detect different aspects of the motion stimuli (36, 37). Vision, for example, cannot distinguish the effect of self-motion from the actual motion in the observed scene (e.g., feelings of illusory movement when looking at a moving train from a window seat) and becomes less reliable as the light dims. Within the vestibular organs, the semicircular canals, working as gyroscopes, inform us when our own angular velocity changes, but they are unable to report constant-velocity rotation; the otolith organs, in turn, measure the direction of accelerations, but, as any accelerometer, they cannot distinguish between gravity and inertial forces (38). In the brainstem and cerebellum, all sensory signals are merged, weighing them according to their reliability in a process optimized to obtain the best estimate of our natural self-propelled motion (Figure 1) (39, 40). However, passive artificial motion (e.g., experienced on a transport system) induces unnatural motion stimuli that lead to combinations of sensory signals judged impossible by our brain. Since each sensory signal could be interpreted as resulting from a different natural motion, our brain has to solve a problem of conflicting information, usually termed the sensory conflict (41–43).

## Theories on Motion Sickness

The sensory mismatch theory (2, 44, 45), the most widely accepted theory on motion sickness, states that conflict of motion stimuli alone is not sufficient to cause motion sickness. This conflict is only perceived as nauseogenic, if the present pattern of the rearranged sensory motion signals is at variance with what is expected from previous experience (2). A mathematical formulation of this theory defines the conflict vector as the difference between all sensed and all expected sensory information (42, 43, 46).

The computation of this conflict vector can be simplified, if one only considers the difference between the subjective vertical and the vertical provided by the sensory signals, as proposed by the so-called subjective vertical conflict theory (47–50). On first consideration, the sensory mismatch theory and the subjective vertical conflict theory appear to be equivalent, but centering the computation of the conflict on the perception of verticality has significant impact on the definition of the nauseogenity of specific stimuli. For example, in the course of an angular on-vertical axis velocity step (i.e., a short acceleration phase followed by constant-velocity rotation), in an illuminated environment, the vestibular and optokinetic (rotatory information detected from visual input) signals are conflicting due to their different dynamics (vestibular: high-pass filter; optokinetic: low-pass filter). According to the classical sensory mismatch theory, this condition should cause motion sickness while it should not according to the subjective vertical conflict theory (51, 52). In reality, even a simple visual stimulus rotating around an Earth-vertical axis might induce motion sickness in susceptible subjects. This, however, does not disprove the subjective vertical conflict theory since, as explicitly stated by the authors (53), motion sickness in these situations is usually weak, has a considerably long latency and could be caused by a minor discrepancy between the rotation axis and true verticality. These discrepancies could be caused by a misperception of the center of rotation (52), a uncertainty in the sensory signals (54) or a minor distortion of the perceived vertical occurring when the head is held still for a prolonged time in a position differing from the exact upright (55, 56).

A theory based on the dynamic properties of the angular vestibuloocular reflex and their dependence on gravitoinertial vector orientation links the theoretical and the physiological framework (57–59). It states that the motion sickness is related to the difference between the yaw eigenvector of velocity storage (60), the mechanism responsible for integrating and coordinating signals for self-motion perception (61–64), and the actual vestibular velocity signal. Although specifically designed for head rotations, this theory often parallels the subjective vertical one as the velocity storage processes the sensed rotational velocity vector to align it with the gravitoinertial vector in order to generate a congruent integrated signal. Its yaw eigenvector, which drives such process by reinforcing the associated component of the rotational vector, is therefore close to the spatial vertical (65). The difference between such vector and the actual vestibular velocity signal is, therefore, identical to the conflict defined by the subjective vertical theory, but its computation can be advantageous when studying certain motion stimuli (e.g., cross-coupling – see The Cross-Coupled Stimulus).

A different computational approach to quantify the sensory mismatch has been presented in a series of work by Holly (66–69). The innovative aspect of this approach stays in that it describes the perceived motion evoked by nauseogenic stimuli on the basis of the law of physics without the need of including any specific physiological characteristic of the sensory system involved. This theory redefines the “conflict vector” splitting it in two 3D vectors, namely, a “twist factor,” computing the difference between the expected and the perceived rotation, and a “stretch factor,” computing the difference between the expected and the perceived translation. The results of the model simulations show that the “stretch factor” is the most relevant factor in causing motion sickness in the majority of the conditions (69). Its magnitude depends on the decomposition of the gravitoinertial acceleration in linear acceleration and gravity, confirming the dominant role of the correct perception of verticality (i.e., of gravity vector) in determining motion sickness. Therefore, it should not be a surprise that the simulations match the predictions of the subjective vertical conflict theory, although Holly’s approach changes the focus from a mismatch of verticality into a mismatch of perceived translation.

A theory offering a radically different perspective has been proposed by Riccio and Stoffregen with the name of ecological theory of motion sickness (70, 71). It states that motion sickness occurs when a combination of motion stimuli trigger postural instability (71–73). In support of this theory, different studies have shown not only correlations of motion sickness with postural instability in nauseogenic environments (74–76) but also of susceptibility to motion sickness and postural sway in the absence of visual cues (77). Similar to the previously discussed ones, this theory proposes a tight connection between motion sickness and verticality perception. Yet, it deems the perturbations of postural stability as the cause of motion sickness genesis, moving the focus from a perceptual to a sensory-motor process (73). The main problem of this theory is, therefore, that it excludes from nauseogenity all the conditions where no active postural stabilization is required, although motion sickness have been observed also when lying or sitting, a setup often used in centrifugation experiments (78, 79). Moreover, the theory assumes that the perceptual process of upright is subordinated to its motor control counterpart, a phenomenon that, although supported by early studies (71, 73), is debated (80). The correlation suggested by the ecological theory of motion sickness may still provide advantages for describing situations focused on tasks related to postural stability in nauseogenic motion environments (81, 82).

Notice that we choose to present the different theories mentioned above without discussing the functional, physiological, or evolutionary reasons why certain motion profiles lead to motion sickness. Physiological and/or evolutionary reasons have been widely debated, in part also in connection with the above-mentioned theories (83). Such discussion is, however, outside the scope of the current review.

## Classifications of Motion Sickness

From the earliest studies of motion sickness, classifications of the stimuli causing motion sickness have been proposed (2, 45, 84). The proposed categorization separates the stimuli based on the source of the conflicting sensory signal or on whether the sensory conflict occurs between two actual signals or for the absence of an expected one. Although multiple examples have been provided (84), assigning a specific stimulus to a single group is not always univocal. This approach has been complementary to the theories discussed in the previous section, aimed at providing unifying explanations to clarify the mechanism of motion sickness, independently of the sensors involved.

Overall, at least two major categories of sensory conflict can be distinguished (45): (1) conflict between angular (semicircular canals) and linear (otolith organs) vestibular input and (2) conflict between visual and vestibular input. Detailed examples for the stimuli of each category have been presented in early work (2, 84). Currently, this classification proves to be useful for focusing on a specific nauseogenic stimulus, reproducing it in a laboratory environment and studying possible countermeasures for practical applications. Yet, a deeper understanding of why it causes motion sickness requires specific modeling provided by the theories discussed above. Once a specific nauseogenic stimulus has been selected or identified by its motion profile and sensory involvement, it is, however, often difficult to link it to the general framework of the theories. This problem is enhanced by the lack of quantitative assessments of motion sickness, which is often assessed subjectively alone with questionnaires (19, 59, 85–88). This complicates comparing motion sickness generated by the different experimental paradigms. A comparative evaluation of the assessment methods is, however, outside the scope of the current review. This review focuses on vestibular-only motion sickness (i.e., category 1) with the aim to present both the characteristic of the most relevant nauseogenic stimuli in this category and the way in which they match the prediction of the above-mentioned theories.

## Vestibular Motion Sickness

Vestibular-only motion sickness is being provoked when conflicts occur among different sensory signals of the vestibular system. These conflicts depend on how signals from the semicircular canals, providing transient head rotation velocity signal, and from otolith organs, detecting the gravitoinertial force vector, are integrated. The basic assumption is that gravity is the only know constant acceleration. Accordingly, segregation of the gravity vector from the inertial vector can be obtained by separation of the slow- and fast-changing component of the gravitoinertial acceleration with the first representing gravity and the second the inertial acceleration [frequency segregation hypothesis (89–91)]. This estimate of the direction of gravity, considered by the brain highly reliable, is also combined with the perceived head rotation derived from the semicircular canals (49, 92), and the agreement of both inputs is essential for a correct estimation of the head orientation in space (93–95). A perceived head rotation that does not match with a corresponding change in gravity direction leads to a sensory conflict that, if iterated, evokes motion sickness.

**Figure 2:**
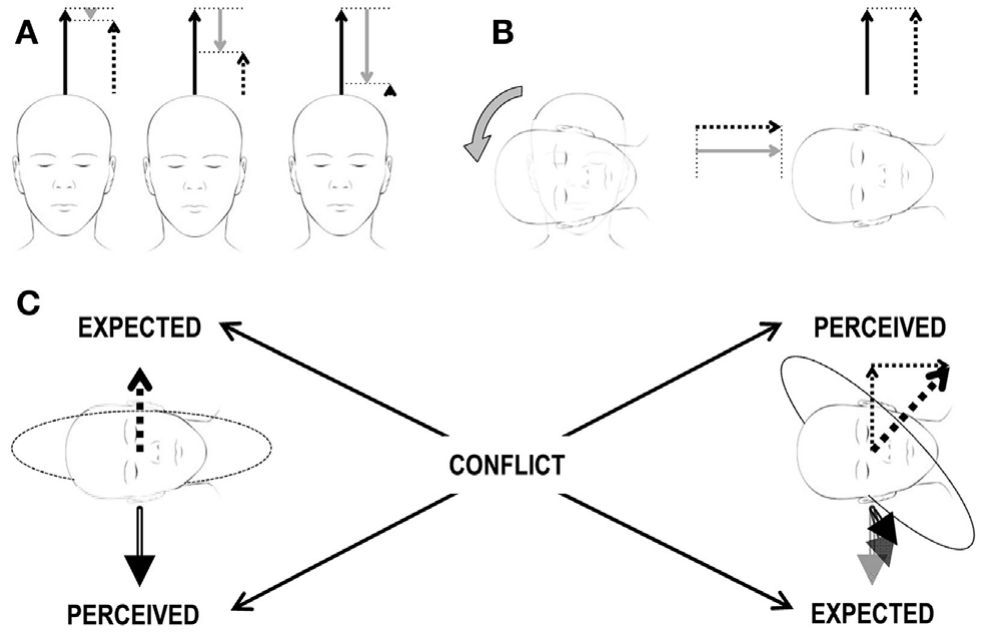
**Scheme of the Coriolis/cross-coupling stimulus and the induced sensory conflict**. **(A)** Constant-velocity rotation. The three heads correspond to three subsequent time points during a constant-velocity rotation. The three arrows above each head correspond to the actual rotational velocity (solid black arrow), to the perceived velocity (dashed arrow), and to the amount of velocity decayed over time (gray arrow) due to the properties of the vestibular system. The sum of the solid black and gray arrows must always correspond to the dashed arrow. In the beginning (leftmost head), the subject has just been accelerated to the constant velocity. The perceived velocity (dashed arrow) is only slightly lower then the actual one (solid black arrow). As time passes (central and rightmost heads), the decayed velocity signal increases and the perceived velocity decreases. **(B)** Head tilt during constant-velocity rotation. Heads and arrows as in **(A)** after the tilt (rightmost head), no actual rotational velocity exists along the head vertical axis (no solid black arrow). However, as the decayed velocity is different from 0, the absence of an actual velocity leads to a perceived velocity different from 0 (recall that sum of the solid black and gray arrows must always correspond to the dashed arrow, so in the absence of a black arrow, the dashed arrow is equal to the gray arrow). The actual velocity is now along the head interaural axis and is perceived by the semicircular canals. The perceived velocity has, therefore, now two orthogonal components. **(C)** Brain expectations and conflict. Leftmost head: the perceived gravity signal (double-line, black arrow below the head) is stable. Accordingly, any expected perceived rotational velocity (dashed arrow above the head) must be aligned with the gravity vector. Rightmost head: the perceived rotational velocity is tilted by 45° (thick dashed arrow above the head) as it corresponds to the sum of the two perceived rotational velocity vectors in **(B)** (thin dashed arrows above the head). Accordingly, the gravity vector (double-line, black arrow below the head) should rotate in the head reference frame as the head changes orientation relative to gravity. Expected and perceived sensory signals do not match, leading to a sensory conflict.

The classic example of this kind of conflict occurs when the semicircular canals perceive head rotations about an axis that is not aligned with the estimated gravity vector, but the latter does not change its orientation relative to the head accordingly. This happens, for instance, anytime we rotate the head about an off-Earth-vertical axis during an ongoing rotation about an Earth-vertical axis (Figure 2). It was first described by Ernst Mach in *Versuch 2* of *einer andern Reihe von Rotationsversuchen*^1^ (96). The second rotation of the head immediately produces *ein eigenthümliches Drehgefühl*.^2^ This additional rotation of the head during ongoing rotation is called Coriolis/cross-coupled stimulus and its impact on the vestibular sensors is referred to as Coriolis/cross-coupling effect (Figure 2 and section The Cross-Coupled Stimulus for details). Repetitive cross-coupling effects are disorienting and nauseogenic (9, 57). The subjective vertical conflict theory, Holly’s physical laws of motion theory, and the velocity storage theory are especially useful to clarify motion sickness evoked by cross-coupled stimuli (68, 97).

Although cross-coupling stimuli are the most known form of vestibular-only conflict, other combinations of vestibular stimuli can elicit motion sickness, even when the canal–otolith conflict is not so explicit. A typical example is provided by linear oscillations, where the absence of rotation may lead to “forgetting” the role of semicircular canals. In these situations, it is likely that their missing activation causes motion sickness (98, 99). The following sections detail these major groups of stimuli inducing vestibular-only motion sickness and discuss their interpretation in the framework of the general motion sickness theories.

### The Cross-Coupled Stimulus

The practical importance of this stimulus was first noted in aerospace medicine (100). When during a sharp turn of a plane, the pilot rotates his head about an axis that is not parallel to the axis of the turn, the resulting stimulus vector acting on the semicircular canals is perpendicular to the plane spanned by the two rotation axes (9). This phenomenon may occur in any curve around an Earth-vertical axis and, it is caused by the inherent decay of the response of semicircular canals to sustained rotations (Figures 1 and 2A). In fact, this decay leads to the computation of a false estimate of angular velocity. The changes of the velocity components occurring while the head is reoriented are, therefore, added to an incorrect starting point (Figure 2B), and the resulting perceived axis of rotation is incorrect, i.e., not aligned with the static gravity vector. Such combination of sensory inputs represents an impossible conflict as any expected perceived rotational velocity vector must be aligned with the gravity vector, if it is stable; otherwise, the gravity vector should rotate in the head reference frame as the head changes orientation relative to gravity (Figure 2C).

Coriolis/cross-coupling may lead to disorientation, motion sickness, and even aircraft casualties (9, 100, 101). Originally the cross-coupled stimulus was referred to as “Coriolis effect,” because the effect was related to the linear movement of the head in a rotating plane (100); later, it was shown that the stimulus can be calculated by the cross-product of the active and passive rotations acting on the semicircular canals (9, 57, 102–104) and was, therefore, referred to as cross-coupling stimulus. In microgravity, the cross-coupled stimulus has the same effect on the semicircular canals, but the disorienting and nauseogenic effect is reduced or even missing (105). This was demonstrated during the Skylab mission M131 (106, 107) where astronauts were capable of repetitively rolling their head while rotating without getting motion sick, while they perceived the same stimulus as nauseogenic on Earth. This finding provides direct evidence that the mechanism underling motion sickness induced by cross-coupling depends on the orientation of the rotation vector with respect to gravity, as predicted by the subjective vertical and the velocity storage theories. The reduced sensitivity to Coriolis/cross-coupling in microgravity implies that in absence of gravity (or any space fixed gravitoinertial force), the illusory rotation signal is not perceived as conflicting as it cannot be integrated and compared with the gravity signal. The conflict is, therefore, missing.

The disorienting and nauseogenic effects of cross-coupled head-roll stimuli depend on the acceleration (direction and magnitude) of the first rotation. If the head is tilted around a roll axis during the acceleration phase of Earth-vertical yaw rotation, the cross-coupling effect is smaller than during the deceleration phase (9). During the acceleration phase, the summed vector of yaw acceleration and cross-coupled pitch acceleration is much closer to gravity than the summed vector of yaw deceleration and cross-coupled pitch acceleration. Hence, the canal–otolith conflict is the largest during yaw deceleration, smallest during yaw acceleration, and in-between during constant-velocity yaw.

Whether head tilts during ongoing yaw rotation are active, e.g., by verbally instructed head roll (9), or passive, e.g., by whole-body tilts on a 3D motorized turntable (108), seems equally disorienting. However, if both cross-coupled rotations are actively performed as part of a natural movement, e.g., during locomotion (109), no disorientation or motion sickness occurs. Guedry and Benson provided a conclusive explanation for this fact (9): during natural movements, cross-coupled rotations always occur during the acceleration phase of a yaw rotation, which minimizes the conflict with the otolith signal; however, cross-coupled rotations during constant-velocity yaw or during deceleration from constant-velocity yaw are not part of natural movement repertoire.

#### Cross-Coupled Rotations, Motion Sickness Theories, and Velocity Storage

All of the above-mentioned theories are able to explain why motion sickness is induced by cross-coupling stimuli. The sensory conflict is indeed evident as semicircular canals signals a rotation axis in conflict with the one suggested by changes gravitoinertial force. By describing how the stimulus should be perceived by the brain according to the law of physics, Holly specifically showed that both stretch and twist factors are different from 0 (68). The direct involvement of the gravity direction in the conflict confirms the central role of verticality perception and matches, therefore, the prediction of the subjective vertical theory (97). Similarly, it was demonstrated that the velocity storage mechanism (60) plays an important role in making the cross-coupled stimulus nauseogenic (58). It is worth to note that in this condition, the subjective vertical theory (47) seems to be experimentally indistinguishable from the velocity storage theory. During a constant-velocity rotation, the yaw axis eigenvector of velocity storage is identical to the spatial vertical, and therefore, the conflict vector computed according to the two theories between such vector and the vestibular velocity signal evoked by the head tilt is identical (see Theories on Motion Sickness for details). However, the latter hypothesis makes the precise prediction that the cerebellar nodulus and uvula, which control the dynamics and 3D kinematics of velocity storage (110–112), are the main central structures involved in motion sickness.

The importance of the time constant of velocity storage in determining the motion sickness sensitivity during paradigms with repetitive cross-coupled stimuli is remarkable (57, 80). During constant-velocity yaw rotation, subjects with short time constants were able to make more head-roll movements before reaching full motion sickness than subjects with long time constants. This can also explain why subjects can be habituated to cross-coupled stimuli (113–115), which is most likely due to the reduction in the velocity storage time constant with repetition (78, 116, 117). The decrease in motion sickness susceptibility by baclofen probably works *via* the same mechanism. Interestingly, the relation between velocity storage time constant and motion sickness appears to have a general validity, as individuals with long time constants are more prone to motion sickness (118, 119), while shortening the time constant pharmacologically or by habituation reduces motion sickness sensitivity (113, 120–122). The role of the velocity storage in determining the nauseogenity of the cross-coupling stimuli can also explain the reduced motion sickness caused by such stimuli in weightless condition (i.e., astronauts or during parabolic flight). The absence of the gravity load on the otolith organs, indeed, deactivates the velocity storage removing, therefore, the source of the conflict (80, 123).

The link between motion sickness and velocity storage has also relevance for the assessment of nauseogenic stimuli through vestibular responses. In the absence of ocular motor deficits, the 3D angular velocity vector of the eyes in total darkness closely reflects the 3D central vestibular velocity signal. This signal represents the sum of the afferent vestibular signal and a partly integrated version of this signal, i.e., the output of the velocity storage (60). While the direct vestibular signal is three-dimensionally isotropic, the velocity storage mechanism in humans acts predominantly in the yaw plane (124–126). Recording eye movements in three dimensions (horizontal, vertical, and torsional) lends itself to study the impact of cross-coupled stimuli on the central vestibular system by analyzing the 3D disparity between head and eye movements (57, 108). Moreover, eye movement parameters, such as gain and time constant of vestibular nystagmus, can be related to measures of motion sickness and to monitor the effectiveness of habituation protocols (78, 79, 118, 121, 127).

### Linear Oscillations

The role of linear oscillation in determining motion sickness was first studied in relation to seasickness, i.e., motion sickness induced by the oscillatory motion of a boat at sea. While some studies were done in real ships and hovercraft (128, 129), others were conducted in simulated condition, isolating the linear from the angular component of the motion (130–132). All these experiments agreed that vertical linear oscillation at low frequency is the most important stimulus in causing seasickness (129). Specifically, any oscillation at frequencies between 0.1 and 0.5 Hz was found to be nauseogenic, with a peak around 0.16 Hz (129, 131–133). Within a given frequency, motion sickness increases monotonically with increasing acceleration (131, 132). More recent studies showed that similar responses are evoked by linear horizontal oscillations in both lateral and fore–aft directions (99, 134–141). By comparing fore and aft and vertical acceleration of similar magnitudes and frequencies, Golding and colleagues found that the first causes stronger motion sickness than their vertical counterpart (99).

The motion sickness induced by linear oscillations may apparently contrast with the sensory conflict theory, since they cause variations of a single sensory signal. Moreover, since high frequency oscillation do not apparently cause disturbances (142), it is clear that not all oscillatory movements cause motion sickness. Physical motion at frequency higher than 1 Hz not only does induce motion sickness but can also be beneficial if added to low-frequency oscillation as it reduces the reliability of the conflicting sensory input (143). The picture becomes clearer when recalling that the conflicts of the sensory mismatch theory are defined as a discrepancy between the brain expectation and the actual arrangement of sensory inputs (2, 42–46). To understand how sensory inputs are processed during linear oscillation, one must take into account that the otolith signal is normally low pass filtered to separate the gravity vector, expected to undergo relatively slow variations in direction and the inertial component of the sensed gravitoinertial vector (frequency segregation hypothesis) (89–91). Accelerations during linear oscillations below a specific frequency are, therefore, added to gravity as the brain fails to identify them correctly and perceives an unexpected change of the gravity vector, unmatched by other sensory signals. The frequency segregation processing is also at the base of the computation of the error vector according to the subjective vertical theory (47–50). To reproduce the differences between horizontal and vertical oscillations, however, the initial theory was extended including an additional internal conflict signal, specific for horizontal linear accelerations [subjective vertical–horizontal theory (SVH)] (144). The SVH theory has been successfully used to predict motion sickness on different vessels at sea (145). Successful prediction of the nauseogenity of linear oscillation at specific frequency can be obtained by applying the theories of Holly (69), with the advantage that the stretch factor represents a linear displacement error resulting from multisensory computation and requires, therefore, no differentiation between vertical and horizontal acceleration conflicts.

#### Combined Linear and Angular Oscillation

In real condition (e.g., for a vessel at sea or on a bus on a curvy road), it is unlikely that the oscillations are limited to a singular dimension. For example, a small boat hit by a sequence of waves oscillates vertically (heave) at the frequency of the coming waves, but it also shows pitch and roll oscillatory motion that depends on its structural characteristic. Since the early studies on motion sickness at sea (3, 128, 129) were conducted on large ships, providing weak pitch-roll stimulation, it was concluded that heave was the sole nauseogenic stimuli at sea. However, to investigate the reasons behind the widely spread belief that travels on small boats are more provocative than large one (146), simulation studies have been specifically designed to investigate whether combination of angular and linear oscillations are more provocative than linear oscillations alone (147). Although earlier studies found no differences (132, 148) when combinations of heave and pitch and roll oscillations were used, an increase in motion sickness was observed in a majority of recent studies (134, 135, 139, 147, 149). Overall, the provocativeness of combination of angular and linear oscillations depends on the specific combination of acceleration and frequency content of the two stimuli (135, 147).

## Example of Vestibular Motion Sickness in Everyday Life

### Motion Sickness in Tilting Trains

Tilting the car bodies of trains compensates for the centripetal acceleration during turns by bringing the vertical axes of the cars closer to the gravitoinertial force vector. As a result, the trains can run faster and the lateral thrusts of centrifugal force on the passengers during turns are decreased. Unfortunately, many passengers in tilting trains develop symptoms of motion sickness, a major problem of modern traffic (4, 150). A detailed understanding of the mechanism underlying motion sickness in tilting trains is essential because only such knowledge allows developing technical solutions for the problem (135).

Tilting trains provide an ideal example of how a real, everyday motion condition that induces motion sickness comprises multiple nauseogenic stimuli. Accurate recording of the linear and angular motion of the cars showed that at least three could be the cause of motion sickness in the train: the centrifugal acceleration mentioned above, the jitters of the car, which shows peaks at 0.5 and 1.7 Hz both in roll rotation and lateral translation (88), and the tilt of train itself, as it provides a typical cross-coupled stimulus with roll movements during both the acceleration and deceleration phases of yaw rotation (88). Various studies showed the nauseogenic role of the lateral linear acceleration in actual and simulated conditions, in particular if combined with roll tilts (99, 135, 137, 139, 150, 151). Accordingly, motion sickness of passengers in tilting trains can be reduced by decreasing the angle of the compensatory roll tilt (4, 88, 152). The usefulness of this approach is limited because the velocity of trains on curves has to decrease, which negates the purpose of the tilt. With respect to the jitter, while 1.7 Hz oscillations are way above the nauseogenic range, 0.5 Hz is likely causing disturbances (see Linear Oscillation). Finally, cross-coupling (see Cross-coupling) is one of the most powerful nauseogenic stimuli; however, whether the relatively low yaw velocities around 4°/s, which are typical on curved tracks, are adequate to produce cross-coupling motion sickness is not known. Interestingly, it has been observed that if subjects’ heads were tilted during lateral acceleration, they had strong motion sickness, but if the head roll was initiated before the lateral acceleration, there was no motion sickness (153). Similarly, by comparing different tilting systems, it has been recently showed that adequate synchronization of roll tilt with changes of yaw velocity on curves, however, eliminates motion sickness (88). These findings clearly suggest that the phasing between the change of yaw velocity and the change of roll position seems to be a decisive factor and confirm that cross-coupling also plays a role in the nauseogenity of tilting trains. Overall, these studies, by analyzing different aspects of the train motion stimuli, underline the agreement between the nauseogenity of an actual motion and the prediction of the theories presented above. The sustained lateral acceleration due to centripetal force causes a tilt of the perceived direction of gravity that depends mainly on the radius of the curve (as the angular velocity of the train is relatively small). If in these conditions, the perceived rotation is further misaligned by a delayed roll tilt, the resulting cross-coupling can be significantly nauseogenic and the amount of conflict can be computed according to the misalignment in the velocity storage vectors, the discrepancy in the perceived and expected subjective vertical, or the stretch and twist factors.

### Motion Sickness in Cars and Public Road Transport

Motion sickness in road vehicle is a very common experience, with high incidence of vomiting, particularly in young passengers between the age of 2 and 12 years (154, 155). By correlating motion sickness with car motions, multiple studies evidenced that the relevant nauseogenic stimuli in road transport are the low-frequency (<0.5 Hz) horizontal linear oscillations (155–157). Relative contributions of fore–aft and lateral accelerations seem to be similar as well as similar are their acceleration spectra in the frequency range relevant for inducing sickness (0.1–05 Hz) (156). The vertical component of linear motion instead has relevant magnitude only between 1 and 2 Hz (142) and, according to the studies on linear oscillation (see Linear Oscillations), does not appear to cause discomfort (99, 158). Hence, motion sickness occurrence in cars and public road transport seems to be mainly influenced by the quality of the driver and the condition of the road (e.g., curvy cross-country versus highway), both factors determining lateral oscillations. The mechanical characteristics of the cars (e.g., active suspension), which control the amount of vertical oscillations, were shown not to play a determinant role (142, 158, 159). The variation of motion sickness with seat position, increasing from the front to the back of the vehicles, can also be explained by the corresponding increase in the magnitude of lateral oscillations (158). The known fact that drivers rarely become motion sick may be due to the driver’s prediction of low-frequency horizontal accelerations as they depend on the driver’s behavior (158, 160, 161). This prediction may allow engaging various compensatory actions, such as tilting the head to align with the tilted gravitoinertia, therefore minimizing the lateral oscillations, a behavior often observed in the drivers (161). Accordingly, motion sickness in rally co-drivers varies considerably as a function of the specific activity, which may involve different levels of interaction with the driving and the environment (162). A recent study, asking the passenger to actively align the head to the gravitointertia, mimicking therefore the driver’s behavior, showed a significant reduction in motion sickness with respect to normal seating with the head upright (163, 164). These considerations are also of great importance for the design of self-driving cars. As suggested by a recent survey on the preferred activity to be done in a self-driving vehicle (165), the benefit of such emerging technology stays in the freedom to disengage from the driving activity and the surrounding environment, therefore increasing the sensory conflict. Any activity causing a strong sensory conflict, however, will be unbearable for motion sickness sensitive individuals and will be partially hindered in more robust ones, reducing the potentially positive impact of this novel technology on the society (166).

## Author Contributions

The authors wrote the review, collected the references by literature search, and edited the final version.

## Conflict of Interest Statement

The authors declare that the research was conducted in the absence of any commercial or financial relationships that could be construed as a potential conflict of interest.

## Footnotes

^1^Translation by Young, Henn & Scherberger (Mach), 2001: another series of rotation experiments.

^2^Translation by Young, Henn & Scherberger (Mach), 2001: strange sensation of turning.
